# Policymakers and the example of smoking to children: A qualitative study

**DOI:** 10.1186/1617-9625-9-1

**Published:** 2011-01-22

**Authors:** Sheena Hudson, George Thomson

**Affiliations:** 1 University of Otago, Wellington, New Zealand

## Abstract

**Background:**

The normality of smoking that children are exposed to is associated with increased risk of smoking uptake. To better understand policymaking that could address this issue, our aim was to identify and document the views of New Zealand policymakers regarding the example of smoking behaviour to children, and the policy responses they preferred.

**Method:**

We analysed public documents for relevant statements, and conducted semi-structured anonymous interviews with 62 purposively selected policymakers. We identified views of New Zealand policymakers regarding: the example to children of adult smoking behaviour, and the policy responses they preferred.

**Results:**

In both public statements and anonymous interviews, policymakers demonstrated that they perceived a clear relationship between the example of smoking and children taking up smoking. However, they showed a general unwillingness to support further smokefree legislation in areas frequented by children. Rather, they preferred to educate adults about their impact as models for youth behaviour.

**Conclusions:**

Health advocates in New Zealand and elsewhere may require more evidence of the effect of relevant legislation and of public support, and wider alliances, to significantly move policies specifically to reduce the example of smoking.

## Background

Children tend to adopt behaviour that they see as normal. The normality and extent of smoking that children are exposed to, such as seeing parents, siblings, and friends smoke, or seeing smoking portrayed in films, is associated with increased risk of smoking among children [1-4]. Other research has found that the frequency with which youth observe smoking is positively associated with their perceptions of the acceptability of smoking,[5] and that social modelling of smoking by peers at school increases the risk of smoking [6,7]. This evidence suggests that decreased examples to children of smoking will decrease the risk of children starting smoking. A frequent reason given by smokers for quitting is so as to not set a 'bad example' for children [8].

The example of visible public or private smoking can be seen as part of the process of 'normalisation' of smoking within society. This can also occur through the wide availability and visibility of tobacco products, the presence of tobacco promotion (in most jurisdictions), and the portrayal of smoking in the media.

The recognition that the example of smoking is a risk factor for children taking up smoking, by the public and policymakers, appears to be an increasing policy driver of new tobacco control policy actions. These include the introduction of explicit information for smokers of the effects on children of the example of their smoking, through warnings on tobacco products. Another response has been the introduction of smokefree outdoor area policies in schools and other settings.

Policies encouraging or requiring outdoor smokefree areas have increased during the last 10 years in North America,[9] Australasia, Hong Kong, Singapore,[10] and elsewhere [11]. This move has been driven by a range of factors, including perceived secondhand smoke risks, litter, annoyance and fire risks [10,12,13]. One of the reasons often given for introducing these policies is to reduce the visibility of smoking to children. For instance, the Queensland Government supports its requirements for outdoor smokefree places with the statement:

'It is important to remember that efforts to help adults to quit smoking and reducing exposure to smoking in public places, sends a positive message to children about not smoking' [14].

Educational authorities in a number of countries have been aware of the role of example in youth smoking. In 1993, a model school policy on smoking was used by the United Kingdom Health Education Authority which stated: 'Children need to receive consistent messages and require non-smoking role models within the school' [15]. The reason for outdoor smoking policies is often described explicitly as reducing example effects of smoking among children. For example, in 2005 the Scottish Government guidelines suggested that:

'Smoke-free policies in external areas frequented by children and young people, like playgrounds ... will help to denormalise smoking further and discourage young people from being influenced by what they may see as an 'adult' activity. Scenes of parental smoking at the entrances to schools, for example, are to be discouraged' [16].

The Australian Government National Childcare Accreditation Council has stated that it is 'important that adults responsible for children model positive and healthy behaviours as children often learn and emulate the actions of adults' [17].

Compared to the genesis of secondhand smoke policies,[18-24] little is known about the thinking by policymakers behind the emergence of the *example *of smoking as a tobacco control policy issue [13]. In order to explore the ways in which policymakers consider the example of smoking to children more in depth, we use the case of New Zealand. This country has comprehensive requirements for smokefree workplace and public interiors, and has required smokefree school grounds since 2004 [25]. Some outdoor stadia enforce smokefree policies. Some local authorities have 'educative' smokefree policies for parks and playgrounds, which rely on publicity and public pressure, rather than bylaws [26]. In a 2007 national survey of New Zealand adults, 76% said it was not at all acceptable to smoke in outdoor children's playgrounds [27]. In a 2007-8 survey of New Zealand *smokers*, 66% disagreed with the statement 'smoking should be allowed at council-owned playgrounds' [28].

Our aim in this study was to identify and document the views of New Zealand policymakers (politicians and senior non-elected officials) regarding the example of smoking behaviour to children, the policy responses they preferred, and how to achieve these policies. We also suggest general preconditions for measures to reduce the example of smoking, which may apply in a range of countries.

## Methods

We gathered qualitative primary data from relevant public documents (including the media) and anonymous interviews. A purposive selection of over 20 websites was searched in March and April 2008, focusing on those most likely to give policymaker statements about the example of smoking and children. The Factiva media database was searched for the New Zealand region for the period since January 1998. The websites explored included: the New Zealand Government http://www.beehive.govt.nz/, Ministry of Health, and the New Zealand Parliamentary Debates (Hansard - http://gphansard.knowledge-basket.co.nz/han/005-01.html). Searchwords included smoke, smoking, smokefree, smoke-free, children, child, infant, youth, home, role-models, modelling, example and normal. We searched for statements by national and local politicians, and by government agencies.

These searches produced 310 documents which included at least a tobacco-related term (eg, 'smoking'), a child-related term (eg, 'children') and an example-related term (eg, 'modelling'). They included annual reports of government agencies such as the Health Sponsorship Council and the Ministry of Health, parliamentary speeches and press releases from 1998 to 2008. Statements from local authority councillors were found in national and regional newspapers. All these documents were screened for policymaker statements about the example of smoking behaviour to children, the policy responses they preferred, and how to achieve these policies.

Additionally, we compiled a list of 88 potential interviewees, of current and past Members of Parliament (MPs), current District Health Board (DHB) board members, and current and past senior central government officials. Inclusion criteria included an interest in health policy and having been in a position to influence health policy within the past five years. For officials this included the ability to give advice to Cabinet Ministers.When approached they also suggested further potential interviewees.

To ensure a wide political spread, we approached 20 National Party MPs and 14 Labour Party MPs (the two dominant parties). In total we approached 48 MPs, 54 officials, and five DHB board members during April 2008 to February 2009 (n = 107). Sixty-two policymakers agreed to be interviewed (22 politicians and 40 officials) giving an acceptance rate of 41% for politicians and 77% for officials. Nine of the MPs were from the 'left' parties, and eight were from the 'centre' or 'right' parties. Almost all the officials had at least 10 years experience in government.

We conducted in-depth anonymised interviews (20-60 minutes long) of policymakers using a semi-structured interview schedule based on previous research experience and knowledge of the literature. We asked participants the core question: "what are your thoughts about how children start to smoke?" This was followed by prompts if areas were not already covered, asking their thoughts about role modelling by parents, other adults, friends, adults outside in public, and media stars.

Interviews were digitally recorded and transcribed verbatim. All interviews, and all documents that included policymaker statements about the example of smoking behaviour to children, the policy responses they preferred, and how to achieve these policies (15 local councillor statements, and 89 national level media items, reports, speeches and media releases) were included in the data analysis. Data were collated and analysed using a Template Analysis approach which involved the development of a coding 'template' [29]. This summarised themes identified by the researcher(s) as important in the data set, and organising them in a meaningful and useful manner. Hierarchical coding was used; broad themes encompassing successively narrower, more specific ones. The prompted questions were used as *a priori *codes; themes expected to be relevant to the analysis, some of which were later modified or dispensed with when they did not prove to be useful or appropriate to the actual data examined.

The analysis proper involved reading through the data, marking any segments that appeared to tell the researcher (s) something of relevance to the research question(s). Where such segments corresponded to *a priori *themes, they were coded as such. Otherwise, new themes were defined to include the relevant material and organised into an initial template which was then applied to the whole data set, and modified in the light of careful consideration of each transcript with the support of computer-assisted techniques. Inter-coder auditing was completed by the themes being discussed and checked against the data by the second author. Once a final version was defined, and all transcripts had been coded to it, the template served as the basis for the development of the salient themes described.

## Results

The documentary evidence indicates that New Zealand central and local government agencies and officials have increasingly focused on the 'example of smoking' issue since 1998. The effect of sporting figures being smokefree, and the normalisation of smokefree activity has been explicitly stated by the Health Sponsorship Council (HSC) since 1997 or before [30]^p.8^. The Associate Minister of Health, Tuariki Delamere in 1998 stated in Parliament:

*'After parents, teachers are the next strongest role models young people have. ... Showing young people that it is possible to lead an active and healthy lifestyle without smoking is a responsibility and challenge I urge all teachers to accept ' *[31].

Since 2003, the HSC annual reports have been more explicit in their statements about the example of smoking. That year, the report mentioned the role modelling of smoking, and HSC efforts so that 'fewer young people see smoking as a social norm' [32]^p.7^. By 2006, the annual HSC report detailed the specific objective of:

*'reducing the number of settings in which young people are exposed to smoking behaviour' and 'promoting not smoking around young children in any setting at any time in order to reduce the likelihood of young people taking up smoking' *[33]^p.4^.

From 2003, there was more specific consideration by parliamentary policymakers of the example of smoking. The Health Select Committee, in arguing for smokefree schools and school grounds, wrote:

*'we consider that the purpose of the legislation includes preventing young people from being influenced by seeing other people smoke in their place of learning' *[34]^p.7^.

The debates around the Smoke-Free Environments Amendment Bill in 2003 revealed some of the thinking behind the ban on smoking at schools. MP Steve Chadwick, sponsor of the Bill in Parliament, said in Parliament:

*'Take away the irony of trying to teach children never to pick up smoking and never to start, but of allowing teachers to smoke on school campuses' *[35].

Policymakers interviewed had similar opinions:

'I thought the sort of thing that influences kids is any significant adult. So it might be a parent, it might be a teacher; it might be a significant adult in that child's life'

From 2006, the idea of smoking as an example of behaviour appeared to have become established in government statements. In October 2006, Associate Minister of Health Damien O'Connor said in a speech:

*'It is crucial that we continue to promote smoke-free homes, cars and public places, as we know it sets a good example to children. ...the more children and young people are exposed to parental smoking in their home and other places the more likely they are to become smokers '*[36].

The interviewees largely supported the direction of government statements. Asked how they thought children started smoking, most stated they believed children copied the smoking behaviour of adults seen around them, especially when seen within the family where it is seen as 'normal or 'adult' behaviour:

'*When a child has been brought up in a smoking house, ...it's the norm, mum and dad smoke, aunties smoke, it's smoking all the time round them'*

### The example of smoking in outdoor areas

Both documentary statements and interview material highlighted the example of smoking outdoors. In 2007, the then Prime Minister Helen Clark was quoted as saying (about smokefree seating areas in the Warriors professional Rugby League club grounds):

*'I see it as a clear demonstration the Warriors are committed to being smokefree role models to their young fans. I would like to encourage other rugby league clubs, and all our sporting groups, to make their grounds smokefree in the interests of a healthy next generation '*[37].

From early 2008, a Ministry of Health website appeared to link smokefree outdoor places with the need to reduce the example of smoking to children:

*'Some councils such as South Taranaki and Upper Hutt have made the decision to make their parks and playgrounds smokefree to help denormalise the behaviour. Research shows the less children see smoking around them, the less likely they are to start' *[38].

Since 2005, there have been a number of statements by local authority elected and appointed officials, on limiting the example of smoking to children [39,40]. For instance, when in 2005 the South Taranaki District Council put in place the first New Zealand smokefree council parks policy, an official was quoted as saying:

*'The purpose of the Policy is to demonstrate ...that a smoke free lifestyle is both desirable and the norm in South Taranaki ... it's about leadership and role modelling. We can appeal to smokers to consider the interests of others and to be positive role models for our children' *[41].

Policymakers interviewed also particularly emphasised that smokefree outdoor places were important:

'In terms of modelling, things like playgrounds become important and sportsgrounds.'

'As role models, adults should not be smoking around play areas.'

### Preferred policy responses

In spite of the strength of their opinions regarding the negative effect of the example of smoking to children, almost all the policy-makers interviewed were extremely hesitant to legislate for smokefree policies in the car, home, sports field or playground. They saw this as the state being overly intrusive in individual adults' private lives, and did not believe the public were ready for it. They believed instead that the education of adults was the most appropriate way to reduce the example of smoking to children:

'I would start with adults ... part of that is to actually ask people if they realise what the messages they are giving to their children...... before you introduce legislation.'

'Whenever something's imposed, people are always trying to find ways around it, or think oh, the government, or the state's interfering too much in my life'.

The National Party (dominant party of the current government) statement was:

*'Although smoking is currently an individual's choice we support ongoing programs and campaigns through education, public pressure and lobbying Parliament to help highlight this issue' *[42].

### How to achieve further smokefree policies

When interviewees were asked how further smokefree policies might be achieved, they had various suggestions, including education in the widest sense. Some suggested that a societal change in attitude to the safety of children was required:

'*We'll be much more effective if we talk about it from a child's safety perspective. And put it in that same bucket of issues, such as don't text on your telephone [while driving],... don't smoke in the car...because it's a safety issue for children.'*

One official describe how this value-based argument might work:

'If you can appeal to those wider social responsibilities - caring, parental responsibility'

Some of those interviewed believed that many adult smokers in New Zealand are not aware of how their smoking behaviour affects children:

'A lot of people don't see themselves as role models ...they don't think that anybody looks at their life and take notice, but they actually are influencing people all around them'

Others were emphatic that extending the voice of young people was an effective strategy to increase smokefree areas. There was wide agreement among policymakers for the need for strong and widespread public support:

'*[The Government] 'can only legislate if there's a strong ground swell of support. ... the chances are that unless that support is there, ... a future government's going to come in and get rid of it.'*

Some policymakers suggested the importance of starting with local councils before taking any further smokefree legislation to national level:

'Work with their local councils, to try and work it through. ...once it becomes more wide spread, perhaps bring it to the government'

One very experienced policymaker noted the importance of local voices, of evidence and the need for a critical mass before politicians will lead new legislation:

'When you've got a critical mass of that [local support], then your politicians would follow. See, politicians will be leaders, but only when the gap is not too big'

## Discussion

The results showed a strong theme of awareness amongst New Zealand policymakers of the significance of the example of smoking in front of children, both in the documentary evidence and in the analysis of interviews. This theme was consistent across both national and local policymakers and through all political parties. Awareness has not necessarily led to effective action. There was also a strong theme of education as policymakers' preferred response to the example of adults smoking. This theme was reflected in current policy. Except for smokefree school grounds and some local authority smokefree stadia policies, government and local authority action has indicated a preference for avoiding legally-enforced smokefree outdoor policies.

The example of smoking to children may not be seen as an immediate hazard, nor is the removal or reduction of examples of smoking seen as currently practical (outside of schools). However, the theme of a wish for education, not regulation, is also reflected in the literature on policy debates about smoking in cars, where the dangers to children are more immediate [20,43].

### Policy implications & recommendations

Better recognition by policymakers in this and other settings of the modelling hazard from smoking could have substantial implications. If seeing or knowing of smoking significantly increases the risk of starting smoking, then part of the answer is to reduce the visibility or existence of smoking. The reduction of population-wide smoking prevalence will always be a primary strategy on reducing the risks from the modelling of smoking [44]. But policies to modify adult smoking in the presence of children are also relevant.

How best to reduce the visibility of smoking in the physical world? The immediate steps include education, social marketing and the use of law. Examples of education and social marketing are graphic messages on cigarette packs, and media campaigns to promote both non-smoking in the presence of children as an accepted norm,[45] and specific smokefree policies in both public and private places where there are children. For instance, the increased adoption of smokefree homes policies by households, and increased knowledge about the dangers of secondhand smoke may increase the adoption of smokefree cars,[46] which in turn reduces the normality of smoking.

Law making and regulation at a number of levels could expand smokefree laws to ensure that all the public areas where children predominate are covered. These could include schools, parks and playgrounds, swimming pool complexes, sports grounds and parts of beaches [11]. Increased smokefree public places may in turn increase the likelihood of smokefree homes [47].

General preconditions for measures to reduce the example of smoking may include evidence of the effectiveness of such measures, and evidence of public support. Our findings suggest that if policymakers, in New Zealand and elsewhere, are to support more smokefree measures specifically to reduce the example of smoking, they may require further evidence of the relevant effects of all options, including smokefree legislation. Evaluation of the existing regulations worldwide for smokefree cars and outdoor areas would help. Examples such as the requirements in California for smokefree playgrounds,[48] in Maine for smokefree parks and beaches,[49] and in Queensland for smokefree beaches, playgrounds and outdoor eating areas,[14] may provide opportunities. However, the evaluation of health promotion role modelling, and its obverse, reduced examples of high risk behaviour, has considerable difficulties [50]. Further evaluating the relationship between general smokefree laws and smoking uptake,[51] may be more productive.

Health advocates may also need to more clearly communicate public opinion to policymakers and the public, and to highlight the issues around the example to children of using a deadly, addictive substance as though it is normal [52]. Repeated surveys of public opinion about smokefree laws,[11,53] may provide supportive trends. They may also need to ally with those concerned with child protection, or with others concerned with the consequences of smoking - eg, litter, annoyance and fires. Where the effects of smoking examples are not of importance to policymakers, the immediate physical effects of secondhand smoke may be easier to communicate. Politically, amplifying the voice of children may be crucial [54]. The careful use of windows of opportunity can be necessary [19,21].

It can be argued that it is a reasonable ethical principle for a society to try to minimise the exposure of children to observing the consumption of tobacco, a highly addictive and hazardous drug. Children are a highly vulnerable population, susceptible to the influences of adult behaviours. Protection from addiction can be considered to be freedom enhancing overall - given that the great majority of smokers regret ever starting [55].

The balance of major relevant ethical considerations - beneficence, non-maleficence, justice, and respect for autonomy,[56] - may be weighted towards increasing smokefree outdoor places if we adopt the principle of putting the protection of children first, and by assessing the balance of benefit over harm [57,58]. The highlighting of ethical obligations for all governments, and legal obligations for nearly all governments, to protect children from harm, provides the basis for national and international action [59].

### Limitations of the study

Recruiting senior politicians was slightly less feasible than expected from previous work,[60] and this may have been because of the impending national election in October 2008. Many stated that they were 'exceptionally busy' as a reason for non participation. Another limitation was the time constraints for some of those who were interviewed, which meant there was less opportunity to explore some of their responses in depth. Nevertheless, given the amount of repetition of common themes, we are confident that the major attitudes to the example of smoking that were held by these policymakers were captured in this study. The anonymity of the interviews generally enabled far more candid statements, compared to those made in public. There are of course the general limitations of qualitative research, including the limited ability to generalise from results, the contestable nature of samples, and diverse understandings of approaches [61-63]. Along with Sandelowski and Barroso, our primary methodological aim has been to make material meaningful for readers, rather than meet particular guidelines [64].

### Further research

This qualitative study could be repeated in other settings. It could be extended with quantitative methods, to investigate for larger and more clearly representative samples, the views found here. In particular, surveys could be used to explore whether policymakers are concerned about the example of smoking, preferred policies to deal with the issue, and how they might achieve the policies.

## Conclusions

Health advocates in New Zealand and elsewhere may require more evidence of the effect of relevant legislation and of public support, and wider alliances, to significantly move policies specifically to reduce the example of smoking.

## Abbreviations

DHB: District Health Board; HSC: Health Sponsorship Council; MP: Member of Parliament

## Competing interests

Although we do not consider it a competing interest, for transparency, GT has undertaken work for health sector agencies working in tobacco control.

## Authors' contributions

Both authors designed the study, helped collect data, both analysed data and wrote the text, both read and approved the final text.
